# Evaluating the Effectiveness of the Supportive Parenting App on Parental Outcomes: Randomized Controlled Trial

**DOI:** 10.2196/41859

**Published:** 2023-01-16

**Authors:** Shefaly Shorey, Evelyn Law, Thilagamangai, Jancy Mathews, Siew Hoon Lim, Luming Shi, Jing Shi Chua, Ruochen Du, Yiong Huak Chan, Thiam Chye Tan, Cornelia Chee, Yap Seng Chong

**Affiliations:** 1 Alice Lee Centre for Nursing Studies Yong Loo Lin School of Medicine National University of Singapore Singapore Singapore; 2 National University Hospital Singapore Singapore; 3 Division of Nursing, KK Women’s and Children’s Hospital Singapore Singapore; 4 National University Polyclinics, Corporate Office Singapore Singapore; 5 Singapore General Hospital Singapore Singapore; 6 Singapore Clinical Research Institute Singapore Singapore; 7 Biostatistics Unit, Yong Loo Lin School of Medicine Singapore Singapore; 8 Yong Loo Lin School of Medicine Singapore Singapore; 9 Mount Elizabeth Novena Specialist Centre Singapore Singapore

**Keywords:** postnatal depression, mobile health technology, social support, COVID-19, psychoeducation, peer support, mobile phone

## Abstract

**Background:**

Adjusting to new or additional parenting responsibilities increases stress and affects parental well-being. Existing research has highlighted both parents’ desire to receive more support. It has also been found that receiving sufficient social support enhances parenting outcomes. With the increasing popularity of mobile health apps, a Supportive Parenting App (SPA) intervention was developed to fulfill the support needs of parents during the perinatal period.

**Objective:**

This study aimed to examine the effectiveness of the SPA on parental outcomes during the perinatal period.

**Methods:**

A 2-group pretest and repeated posttest randomized controlled trial was conducted wherein 200 couples (N=400 mothers and fathers) were recruited from 2 public health care institutions in Singapore. Parents were randomly assigned to intervention (100/200, 50%) or control (100/200, 50%) groups. The SPA intervention consisted of a mobile app–based psychoeducation and peer support program to support parents from pregnancy to 6 months post partum. The outcome measures included postnatal depression, anxiety, parental bonding, parental self-efficacy, perceived social support, and parenting satisfaction. Data were collected at baseline (at >24 weeks of gestation—age of viability in Singapore) and at the first, second, fourth, sixth, ninth, and 12th month post partum. Linear mixed models were used to compare parental outcomes between the groups, and a linear mixed model for repeated measures was used to examine within-group changes.

**Results:**

Parents in the intervention group mostly showed better outcomes compared with those in the control group. Parents in the intervention group had higher perceived social support than those in the control group at the first (effect size=1.59, 95% CI 0.38-2.80; Cohen standardized effect size=1.31; *P*=.01), second (effect size=1.98, 95% CI 1.09-2.88; Cohen standardized effect size=2.21; *P*=.003), and fourth (effect size=2.57, 95% CI 1.62-3.51; Cohen standardized effect size=2.72; *P*=.048) months post partum. However, parents in the intervention group showed significantly poorer parental bonding (effect size=1.67, 95% CI 0.24-3.11; Cohen standardized effect size=1.16; *P*=.02). The other parental outcomes did not differ significantly between groups. The scores of mothers and fathers also differed significantly for all outcomes except parental self-efficacy.

**Conclusions:**

Parents in the intervention group generally fared better, especially regarding perceived social support. However, the lack of statistical significance in most outcomes showed the limited effectiveness of the SPA intervention, which may be because of the COVID-19 pandemic. Parental differences in outcome scores suggest that mothers and fathers have different support needs; therefore, interventions should be tailored accordingly. Further improvements and evaluations are needed to examine the effectiveness of the SPA intervention in enhancing parental outcomes. Despite statistically insignificant results, limitations should be considered to further improve mobile health app–based interventions such as SPA, as they could serve as reliable and convenient sources of support for parents.

**Trial Registration:**

Clinicaltrails.gov NCT4706442; https://clinicaltrials.gov/ct2/show/NCT04706442

## Introduction

### Background

Despite the excitement of having a new addition to the family, most parents, especially new parents, face a range of difficulties that can undermine their well-being and capacity to care for the newborn. Changes in one’s social life, adjusting to new parent identity, and taking on newborn care tasks can often cause parents to feel stressed and lead them to experience a loss of individuality as well as couplehood [1,2]. In addition, several studies have shown that most parents reported receiving insufficient support during the perinatal period, affecting their transition to parenthood [3-5]. Insufficient support during the perinatal period often affects the mother’s well-being and recovery during the postpartum period [6]. In contrast, fathers have expressed their desire to be more involved in perinatal care, hoping to receive more informational support to enhance their parenthood experience [7]. As recent advancements have led to the emergence of various mobile health (mHealth) technologies [8-10], it could be helpful to use these technologies to provide parents with the appropriate support they need during the perinatal period.

Previous research has found that receiving sufficient social support improves parental and child outcomes [11-14]. Parents receiving more social support tend to engage in more positive parent-child interactions [11], have better parenting skills, and have greater self-efficacy [12]. Mothers who perceived greater availability of social support reported feeling more confident in their competence to cater to the needs of their child [12]. Knowing that help is readily available enables parents to make more effort for developing better parenting skills. Qualitative studies have also revealed that various sources of instrumental, emotional, and informational support play an important role in helping new parents transition to parenthood [14,15]. In contrast, lack of support significantly affects the ability of parents to cope with and adjust to the influx of responsibilities they face when welcoming a new baby into their life [3]. Ong et al [4] explained that most new mothers, regardless of their background, are vulnerable to emotional distress and postnatal depression (PND). Fathers have also reported facing emotional distress, especially because of the confusion regarding their role and involvement in childcare and pregnancy [3]. To obtain more support, an increasing number of parents are turning to more immediate sources of information on websites and web-based communities for their informational and emotional support needs [16]. Parenting websites have thus been described as platforms where parents can shop, socialize, and study.

However, there is a rising concern regarding the reliability of parenting-related content on the internet [17]. A recent study [17] conducted in 2019 found that although pregnancy- and parenting-related information available on the internet was fairly reliable, there was at least one inaccurate source within the top 5 results shown for each topic. Most parents recognize the possibility of encountering inaccurate information on the internet and often attempt to prevent trusting unreliable sites by using only reputable sources of information and cross-checking across various sources [18]. Nonetheless, studies examining the support needs of parents have reported that reliable information on pregnancy and childcare provided by health care professionals is desired [3,4]. Parents hope to receive localized information tailored to their individual needs and relevant to their settings [3,19]. Thus, it is important to develop technology-based interventions that would allow parents to receive accurate information and social support. mHealth apps have been growing in popularity, especially those for pregnancy and parenting [20]. Many mHealth apps boast about providing various useful functions such as providing parenting information, tracking developmental milestones of babies, and allowing for the sharing of photos and videos on the web. Existing studies have found that mHealth apps have various benefits, including improving parenting self-efficacy, increasing parenting satisfaction, and reducing postpartum depression and anxiety symptoms [10,21]. Although these apps can make parenting easier and more exciting, the market is saturated with a mix of good- and low-quality apps that often require parents to undergo cycles of trial and error to find one that suits them [20]. Virani et al [20] found that although evidence-based apps are scientifically accurate and reliable, they often lack the element of user engagement that draws parents into using them. In contrast, although commercial parenting apps tend to provide content that is influenced by profits, they have more intuitive user interfaces that make them more popular.

Given the pros and cons of existing mHealth apps, the theory- and evidence-based Supportive Parenting App (SPA) was developed to support new parents across the perinatal period.

### Aims and Hypotheses

This study aimed to examine the effectiveness of the SPA intervention on parental outcomes such as PND, anxiety, parental self-efficacy, perceived social support, parental bonding, and parental satisfaction across the perinatal period. It was also hypothesized that the intervention group will have significantly lower scores on PND (primary outcome) and anxiety compared with the control group and significantly higher scores for parental self-efficacy, perceived social support, parental bonding, and parental satisfaction compared with the control group receiving standard care from baseline to 12 months postpartum.

## Methods

### Study Design

This study adopted a 2-group pretest and repeated posttest randomized controlled trial (RCT) design. This study was conducted from February 2020 to July 2022. Shortly after the study began, the COVID-19 pandemic broke out in Singapore, leading to the implementation of various safety restrictions. Before recruitment, a statistician involved in the study developed a randomization list using a permuted block randomization method with the Research Randomizer [22] (stratified by hospitals and depression scores—≥9 on the Edinburgh Postnatal Depression Scale [EPDS]). Couples were allocated to either intervention or standard care using sealed envelopes containing nonduplicated numbers (1-200) to determine their allocation to the 2 groups.

### Eligibility Criteria

Heterosexual married couples were recruited from 2 public health care institutions in Singapore based on the following inclusion criteria: (1) both parents were aged ≥21 years, (2) able to read and speak English, (3) had a low-risk pregnancy at >24 weeks of gestation (age of viability in Singapore), and (4) owned a smartphone with internet access. Participants were excluded from the RCT if they were diagnosed with physical or mental disorders that would affect their ability to participate or if they had a high-risk pregnancy (eg, placenta previa major, preeclampsia, or pregnancy-induced hypertension). Those who had a complicated assisted delivery, stillbirth, gave birth to a newborn with congenital issues, or whose infant was admitted to the neonatal intensive care unit were also excluded from the study, as information provided on the SPA might be misleading or unsuitable for them.

### Sample Size Calculation

Previous research has shown that psychosocial and educational interventions implemented during the perinatal period often result in a medium effect size on outcome variables such as depression, anxiety, stress, and self-efficacy [23-26]. Therefore, considering the medium-sized effect of SPA on the outcome variables, postulating Cohen *d* of 0.5 with 90% power and a .05 significance level (2-sided), 85 subjects for each arm were required [27]. Factoring an attrition rate of 20% (based on a previous study [28]), 200 couples were recruited and randomized equally to the 2 groups.

### Intervention

**Figure 1:**
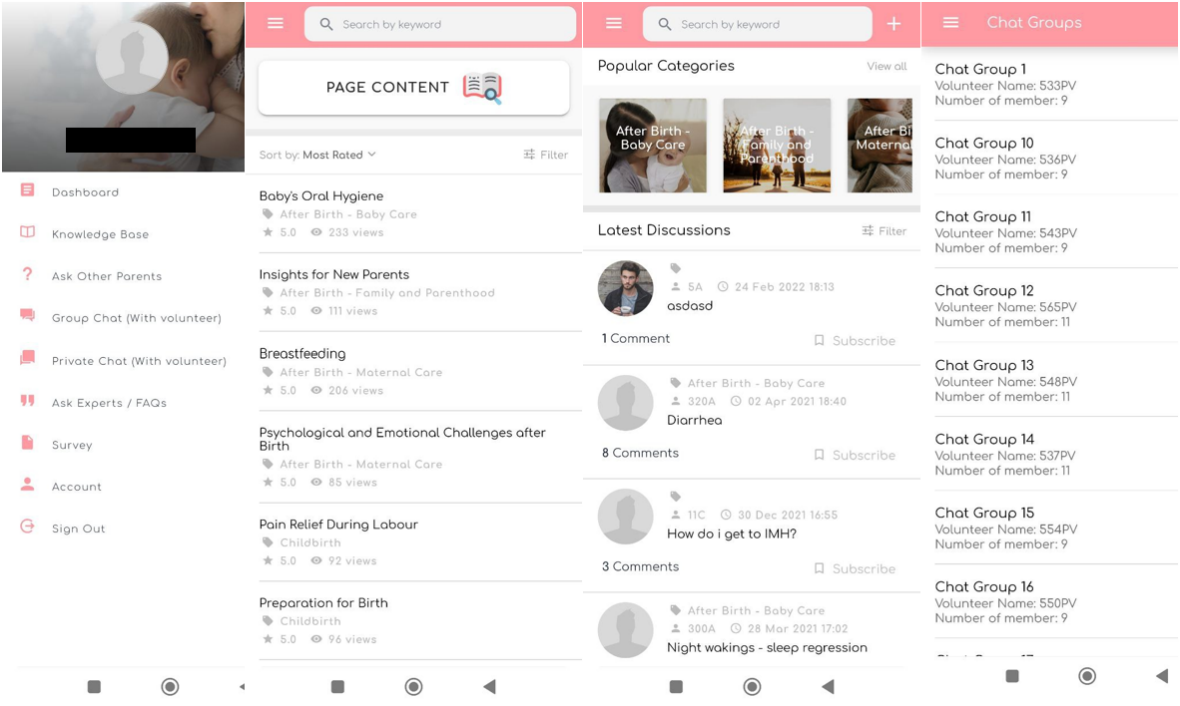
Supportive Parenting App design and features.

Couples assigned to the control group received standard perinatal care provided by the hospitals, consisting of antenatal checkups, optional antenatal educational classes, in-hospital care, and 1 postnatal review 6 weeks post partum. Obstetricians, nurses, lactation consultants, and neonatologists provided perinatal care. In addition to standard care, parents in the intervention group were given access to the theory- and evidence-based psychoeducational mobile app SPA. As part of the SPA intervention, parents in the intervention group were also matched with and introduced to peer volunteers who provided them with emotional and informational support. Well-established theoretical frameworks have guided the development of the SPA, such as the mHealth user engagement pyramid by Singh et al [29], the social cognitive theory founded by Bandura [30], and the attachment theory proposed by Bowlby [31]. These frameworks helped determine the type of information and the mode in which the information should be delivered to fulfill the informational support needs of parents. The parents in the intervention group also received peer support from other experienced mothers who were trained by the research team from the time of their recruitment during pregnancy (baseline—after 24 weeks of gestation) to 6 months post partum. Unique usernames (to conceal parents’ identities) and passwords were used to create an account on the SPA. The SPA included knowledge-based content curated by a multidisciplinary team of health care professionals on pregnancy, childbirth, baby care, maternal care, family, parenthood, and mental health to offer parents accurate, relevant, and reliable information (Figure 1). The content was presented in various forms such as written articles, audio clips, and videos. Discussion forums, frequently asked questions, and expert advice (on daily basis) were also available on the app to resolve any queries that parents might have. To enhance the use of the SPA, frequent app notifications were sent to the parents to suggest timely resources based on the specific needs and major milestones during their perinatal timeline. Peer volunteers and parents would communicate with one another through the mobile app or their preferred messaging platforms. The peer volunteers were specially trained to provide peer support and had previously experienced and recovered from PND. Specific details of the SPA intervention can be found in a published developmental study [32].

### Procedure

Couples were approached by a research assistant during their routine antenatal checkups at 2 public health care institutions in Singapore and provided a summary of the purpose and details of the study. Interested couples were screened for eligibility, and their written informed consent was obtained. Subsequently, they completed a demographic form and a baseline questionnaire. The couples were then randomly allocated to the intervention or control group using an opaque envelope. Participants in the intervention group were given access to the SPA and contacted by their assigned peer volunteer shortly after recruitment. Where possible, peer volunteers were matched with the participants according to their demographic characteristics, such as age. Couples assigned to the control group received only the standard care provided by the hospital. After childbirth, couples from both groups were contacted to record their newborn’s date of birth and other birth details (eg, delivery method and any abnormalities with the newborn) so that their continuation in the project could be ensured based on the selection criteria. Parents also had to enter their birth details into the SPA so that they could receive specific and relevant information pertaining to the respective time points post partum.

Postbaseline data were collected at the following time points for all parents by a different research assistant: (1) 1 month post partum, (2) 2 months post partum, (3) 4 months post partum, (4) 6 months post partum, (5) 9 months post partum, and (6) 12 months post partum. Links to web-based questionnaires were sent to the participants via WhatsApp at each time point, and home visits were scheduled during the sixth month and 12th month post partum to assess the development of their baby (results of child-related outcomes will be analyzed and published separately).

### Outcome Measures

#### Overview

The primary outcome (maternal PND) and secondary outcomes (anxiety, parental self-efficacy, perceived social support, parental bonding, and parenting satisfaction) were measured using validated self-reported questionnaires. Cronbach α was used to indicate the internal consistency of each instrument. The following instruments were used to measure the outcome variables: the EPDS, State-Trait Anxiety Inventory (STAI), Parent-to-Infant Bonding Questionnaire (PIBQ), Parenting Efficacy Scale (PES), Perceived Social Support for Parenting (PSSP), and What Being the Parent of a Baby is Like (WPBL).

#### Edinburgh Postnatal Depression Scale

PND among parents was measured using the 10-item EPDS [33]. Each item was answered on a 4-point Likert scale. The total scores of the EPDS ranged from 0 to 30, with higher scores indicating that the parent had a higher risk of developing PND. Previous research has suggested that a cutoff score of 9 to 12 indicates possible PND symptoms, whereas scores >12 or 13 indicate PND [34]. Cronbach α of the EPDS in this study showed high internal consistency (α=.848), similar to that of a previous study on Singaporean parents [10] where Cronbach α ranged from .811 to .853.

#### State-Trait Anxiety Inventory

The STAI [35] consisted of 2 subdomains, state anxiety (STAI-Y1) and trait anxiety (STAI-Y2), and 40 items (α=.962). Previous studies [10,28] also showed high Cronbach α values (α=.923-.980) for the STAI. Items were answered on a 4-point Likert scale, and the total score ranged from 20 to 80 on both subscales. Higher scores indicate greater anxiety, and the scores are classified such that a score of 20 to 37 indicates no or low anxiety, 38 to 40 is classified as moderate anxiety, and 45 to 80 indicates high anxiety.

#### Parent-to-Infant Bonding Questionnaire

The PIBQ [36] was used to measure parental bonding. It has 8 items, scored on a 4-point Likert scale ranging from 0 (“very much”) to 3 (“not at all”). The internal consistency of the PIBQ was 0.690. Total scores ranged from 0 to 21, and higher scores on the PIBQ suggest poorer parent-infant bonding.

#### Parenting Efficacy Scale

Parenting self-efficacy was measured using the PES [37], a 10-item questionnaire answered on a 4-point Likert scale ranging from 1 (“not good at all”) to 4 (“very good”). This scale has high internal consistency (α=.912). The total scores ranged from 10 to 40, with higher scores on the PES indicating that the parent had a higher level of perceived self-efficacy.

#### Perceived Social Support for Parenting

The 8-item PSSP [38] was used to measure parents’ satisfaction with the social support they received from their partners and others during the perinatal period. Each item was rated on a 5-point Likert scale, ranging from 1 (“very dissatisfied”) to 5 (“very satisfied”). Cronbach α of the PSSP in this study was .875. Participants answered each item on a 5-point Likert scale. The total scores ranged from 8 to 40, with higher scores indicating more perceived social support.

#### What Being the Parent of a Baby Is Like

WPBL [39] comprises 11 items, with each item having a 10-point semantic differential scale ranging from 0 to 9. It had high internal consistency (α=.949). A higher WPBL score indicated greater parenting satisfaction, and the total score ranged from 0 to 99.

### Data Analysis

All analyses were performed using SPSS (version 27; IBM Corp [40]), with statistical significance set at *P*<.05. Descriptive statistics for numerical variables were presented as mean (SD) and n (%) for categorical variables. A mixed model for repeated measures from baseline to 12 months post partum was used to investigate the time trend and time×intervention interaction of the outcome measures over the study period. A linear mixed model with couple identification as a random factor was used to investigate the effect of the intervention on the outcome measures of interest. The above mentioned analyses accounted for demographic covariates, including sex, education, and household income.

### Ethical Considerations

Ethics approval was obtained from the National Health Group Domain Specific Review Board (2019/00875). All participants were informed about the study details and procedures before providing their written consent. The collected data were coded and anonymized. Participation was strictly voluntary, and participants were informed about their right to withdraw from the study at any point without any consequences.

## Results

### Overview

**Figure 2:**
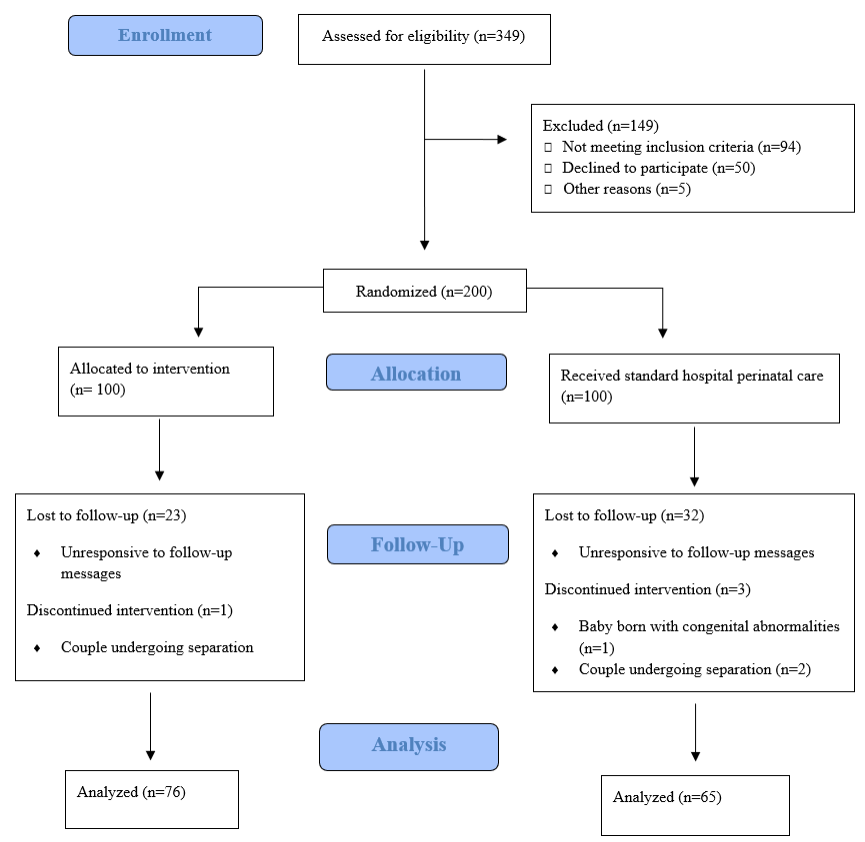
CONSORT (Consolidated Standards of Reporting Trials) flow diagram. EPDS: Edinburgh Postnatal Depression Scale; PES: Parenting Efficacy Scale; PIBQ: Parent-Infant Bonding Questionnaire; PSSP: Perceived Social Support for Parenting; STAIY1: State-Trait Anxiety Inventory (State anxiety); WPBL: What Being the Parent of a Baby is Like.

A total of 200 couples (N=400) were recruited for the study and randomly assigned to the control (200/400, 50%) and intervention (200/400, 50%) groups. The demographic characteristics of all participants are presented in Table 1. The mean age of the participants was 31.4 (SD 4.85) years. Most of the participants were Malay (155/400, 38.8%) and Chinese (143/400, 35.8%). A large proportion (188/400, 47%) of the participants were university graduates, and the gender distribution of university graduates in our sample was similar to that of the national population. Most (304/400, 76%) parents were employed full time and had a monthly household income of over SGD $5000 (US $3700.41; 145/400, 36.2%). According to the parents’ activity on the SPA, the knowledge base content was the most highly accessed component of the mobile app. More specifically, parents tended to view information regarding tips and challenges of caring for their newborns as well as information about breastfeeding. The expert advice column was also popular among parents, with >200 queries submitted. However, this discussion forum has not been widely used. Figure 2 shows the CONSORT (Consolidated Standards of Reporting Trials) flowchart of this study. The average attrition rate at each time point was 28.5% (Table 2). The attrition rate was higher in the control group at all time points. Parents who did not complete all follow-up questionnaires were included in the analyses.

**Table 1:** Demographic characteristics of couples (N=200).

Demographic characteristics				Intervention group (n=100)		Control group (n=100)
**Age (years), mean (SD)**						
	Mothers			29.9 (4.2)		30.5 (4.2)
	Fathers			32.1 (4.9)		33.3 (5.4)
**Ethnicity, n (%)**						
	**Mothers**					
		Chinese	34 (34)		34 (34)	
		Malay	39 (39)		42 (42)	
		Indian	20 (20)		12 (12)	
		Others	7 (7)		12 (12)	
	**Fathers**					
		Chinese	38 (38)		37 (37)	
		Malay	34 (34)		40 (40)	
		Indian	20 (20)		14 (14)	
		Others	8 (8)		9 (9)	
**Educational level, n (%)**						
	**Mothers**					
		Primary school	0 (0)		0 (0)	
		Secondary school	14 (14)		10 (10)	
		ITE^a^ or polytechnic or junior college	30 (30)		42 (42)	
		University	56 (56)		48 (48)	
	**Fathers**					
		Primary school	0 (0)		3 (3)	
		Secondary school	20 (20)		12 (12)	
		ITE or polytechnic or junior college	35 (35)		46 (46)	
		University	45 (45)		39 (39)	
**Employment status, n (%)**						
	**Mothers**					
		Self-employed	5 (5)		5 (5)	
		Full-time employee	68 (68)		67 (67)	
		Part-time employee	2 (2)		2 (2)	
		Unemployed	25 (25)		26 (26)	
	**Fathers**					
		Self-employed	9 (9)		11 (11)	
		Full-time employee	87 (87)		82 (82)	
		Part-time employee	3 (3)		2 (2)	
		Unemployed	1 (1)		5 (5)	
**Monthly household income (Singapore dollar), n (%)**						
	<$1000 (US $740.08)			14 (7.1)		7 (3.6)
	$1000-$3000 (US $740.08-$2220.25)			44 (22.3)		59 (30.4)
	$3000-$5000 (US $2220.25–$3700.41)			62 (31.5)		60 (30.9)
	>$5000 ($3700.41)			77 (39.1)		68 (35.1)
Length of marriage, mean (SD)				4.0 (2.7)		4.1 (3.1)

^a^ITE: Institution of Technical Education.

**Table 2:** Attrition rate across all time points (N=400).

Time points	Intervention group, n (%)	Control group, n (%)	Total, n (%)
First month	24 (12)	49 (24.5)	73 (18.3)
Second month	37 (18.5)	58 (29)	95 (23.8)
Fourth month	58 (29)	68 (34)	126 (31.5)
Sixth month	55 (27.5)	76 (38)	131 (32.8)
Ninth month	50 (25)	84 (42)	134 (33.5)
12th month	44 (22)	81 (40.5)	125 (31.3)

### Main Analysis

The mean parental outcome scores for the control and intervention groups are presented in Table 3. A linear mixed model was used to compare differences in parental outcomes between the 2 groups. There were no significant differences between the control and intervention groups for any parental outcome at baseline. Parents in the intervention group had significantly higher PSSP scores than those in the control group at the first (effect size=1.59, 95% CI 0.38-2.80; Cohen standardized effect size=1.31; *P=*.01), second (effect size=1.98, 95% CI 1.09-2.88; Cohen standardized effect size=2.21; *P=*.003), and fourth (effect size=2.57, 95% CI 1.62-3.51; Cohen standardized effect size=2.72; *P=*.048) month post partum. This shows that parents in the intervention group perceived more social support. However, the PIBQ scores were found to be significantly different between the intervention and control groups at 6 months post partum (effect size=1.67, 95% CI 0.24-3.11; Cohen standardized effect size=1.16; *P=*.02), where parents in the intervention group scored higher on the PIBQ. This indicates poorer parent-infant bonding. The scores on the EPDS, STAI, PES, and WPBL were not significantly different between the 2 groups across all time points, even after adjusting for covariates.

**Table 3:** Mean and SD of parental outcome scores from baseline to 12 months postpartum.

	Baseline, mean (SD)			First month, mean (SD)			Second month, mean (SD)			Fourth month, mean (SD)			Sixth month, mean (SD)			Ninth month, mean (SD)			12th month, mean (SD)	
	Control	Intervention	Control		Intervention	Control		Intervention	Control		Intervention	Control		Intervention	Control		Intervention	Control		Intervention
EPDS^a^	7 (5)	7 (5)	8 (5)		8 (5)	7 (5)		7 (4)	7 (5)		7 (5)	6 (5)		6 (5)	6 (5)		6 (5)	6 (4)		6 (5)
STAI-Y1^b^	39.24 (10.11)	39.34 (9.67)	39.37 (11.45)		37.86 (10.90)	37.46 (11.70)		37.63 (9.76)	35.25 (10.39)		34.38 (10.44)	36.14 (10.88)		35.39 (10.80)	33.95 (10.87)		35.13 (12.30)	34.83 (12.31)		34.59 (11.23)
STAI-Y2^c^	39.67 (8.57)	39.25 (9.05)	40.98 (9.83)		39.11 (9.67)	38.69 (10.92)		37.01 (10.06)	37.28 (10.45)		36.68 (10.89)	37.24 (11.54)		36.90 (11.03)	36.12 (10.61)		36.91 (11.29)	36.47 (11.33)		36.94 (11.08)
PIBQ^d^	3.51 (3.11)	3.56 (3.49)	3.39 (3.17)		3.14 (2.96)	2.85 (2.90)		3.26 (2.80)	3.71 (3.74)		3.73 (4.08)	2.66 (2.81)		4.24 (5.63)	2.78 (2.85)		3.47 (3.59)	2.50 (2.66)		3.01 (2.91)
PES^e^	29.37 (5.56)	29.60 (4.98)	28.09 (5.34)		28.55 (4.95)	29.71 (5.15)		30.31 (4.78)	31.36 (4.89)		31.65 (5.29)	31.60 (5.26)		32.23 (4.61)	32.06 (4.50)		32.40 (4.80)	32.50 (4.57)		32.69 (4.46)
PSSP^f^	12.64 (4.70)	12.74 (4.57)	12.93 (5.07)		14.44 (5.35)	11.82 (5.27)		13.98 (5.27)	11.83 (5.30)		13.20 (5.51)	11.81 (4.78)		12.57 (5.73)	11.62 (5.00)		12.39 (5.97)	11.28 (5.25)		12.55 (5.40)
WPBL^g^	82.45 (12.96)	83.19 (12.16)	80.55 (13.33)		80.85 (14.71)	82.35 (13.49)		83.20 (16.50)	85.84 (11.43)		85.92 (14.90)	86.52 (12.95)		87.04 (9.88)	86.73 (11.10)		86.63 (12.46)	88.55 (9.05)		88.13 (10.36)

^a^EPDS: Edinburgh Postnatal Depression Scale.

^b^STAI-Y1: State-Trait Anxiety Inventory state anxiety.

^c^STAI-Y1: State-Trait Anxiety Inventory trait anxiety.

^d^PIBQ: Parent-to-Infant Bonding Questionnaire.

^e^PES: Parenting Efficacy Scale.

^f^PSSP: Perceived Social Support for Parenting.

^g^WPBL: What Being the Parent of a Baby is Like.

**Figure 3:**
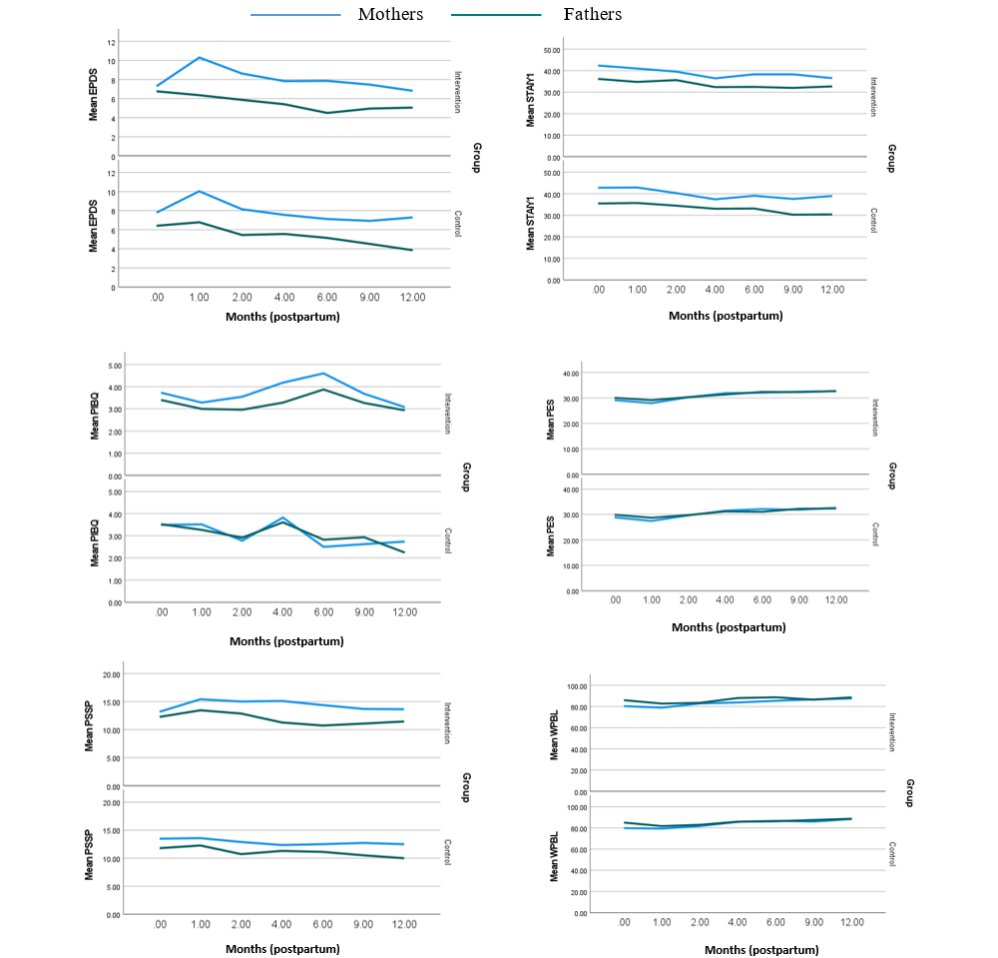
Changes in mean outcome scores of the mothers and fathers in the intervention and control groups from baseline (>24 weeks of gestation) to 12 months postpartum. EPDS: Edinburgh Postnatal Depression Scale; PES: Parenting Efficacy Scale; PSSP: Perceived Social Support for Parenting; PIBQ: Parent-to-Infant Bonding Questionnaire; STAI-Y1: State-Trait Anxiety Inventory state anxiety; WPBL: What Being the Parent of a Baby is Like.

A linear mixed model for repeated measures was used to examine the interaction between time and exposure to the intervention. Figure 3 shows the overall trends of parental outcomes from baseline to 12 months postpartum for mothers and fathers in both groups. From baseline (during pregnancy—after 24-week gestation) to 1 month post partum, both groups showed an increase in EPDS scores (higher depressive symptoms experienced), with a significant number of participants from both groups scoring >9 (132/400, 33%). However, from 1 to 4 months post partum, the EPDS scores for parents in the control group decreased to a larger extent, although both groups had similar mean scores at 6 months post partum. The STAI graph, as well as the mean values in Table 2, showed that the intervention group parents had slightly lower anxiety scores compared with the control group parents until 6 months post partum. Parent-infant bonding, as depicted by the PIBQ graph, was found to be poorer among parents in the intervention group than in the control group (higher scores indicate poorer bonding), where the PIBQ scores steadily increased until 6 months post partum before declining. The PES scores of both groups were highly similar and increased over time. Both groups maintained high levels of parental self-efficacy, but the parents in the intervention group had slightly higher PES scores (Table 2). The PSSP graph in Figure 3 shows that the parents in the intervention group consistently perceived having higher social support than the parents in the control group. Similar to the PES trend graph, the WPBL graph showed that the parents in the intervention and control groups had almost equally high mean WPBL scores at all time points. Hence, all parents reported high levels of satisfaction. Although there were no significant differences in the time trends between the intervention and control groups, the SPA intervention appeared to have generally positive effects on the parents in the intervention group.

### Subgroup Analyses

Results from the analysis of the linear mixed model for repeated measures revealed that the EPDS, STAI-Y1, STAI-Y2, PIBQ, PSSP, and WPBL scores differed significantly between mothers and fathers, regardless of whether they were in the control or intervention groups (Table 4). As seen in the EPDS graph (Figure 3), mothers tended to have higher EPDS scores than fathers (effect size=2.58, 95% CI 1.79-3.37; Cohen standardized effect size=6.46; *P*<.001), regardless of which group they were in. This showed that mothers experienced more depressive symptoms than fathers. The EPDS scores of mothers in the intervention group decreased over time after 1 month post partum, whereas those of mothers in the control group started to increase after 9 months post partum. However, fathers in the intervention group scored higher on the EPDS 6 months post partum, when the intervention ended. Similarly, mothers showed higher levels of anxiety compared with fathers (effect size=6.45, 95% CI 4.45-8.45; Cohen standardized effect size=6.35; *P*<.001), as shown in the STAI-Y1 trend graph. Fathers in the intervention group tended to report better parent-infant bonding, as they scored lower on the PIBQ, compared with mothers (effect size=0.60, 95% CI 0.11-1.10; Cohen standardized effect size=2.38; *P*=.02), although the general trend for both parents was quite similar. In contrast, mothers had higher PSSP scores than fathers in both the control and intervention groups (effect size=2.17, 95% CI 1.31-3.02; Cohen standardized effect size=4.98; *P*<.001), indicating that mothers perceived receiving more social support. The difference in perceived social support between mothers and fathers was greater in the intervention group compared with the control group, as shown in the PSSP graph in Figure 3. Finally, fathers scored higher on the WPBL questionnaire compared with mothers (effect size=−4.38, 95% CI −6.71 to −2.05; Cohen standardized effect size=−3.71; *P*<.001), indicating that fathers had higher parental satisfaction.

**Table 4:** Comparison of outcome scores between mothers and fathers.

	Baseline, mean (SD)			First month, mean (SD)			Second month, mean (SD)			Fourth month, mean (SD)			Sixth month, mean (SD)			Ninth month, mean (SD)			12th month, mean (SD)			Trend difference (baseline as reference)	
	Mothers	Fathers	Mothers		Fathers	Mothers		Fathers	Mothers		Fathers	Mothers		Fathers	Mothers		Fathers	Mothers		Fathers	Effect size (95% CI)		*P* value
EPDS^a^	8 (6)	7 (5)	10 (4)		7 (4)	8 (5)		6 (5)	8 (4)		5 (4)	8 (5)		5 (5)	7 (5)		5 (5)	7 (5)		5 (4)	2.58 (1.79 to 3.37)		<.001
STAI-Y1^b^	42.59 (8.97)	35.83 (9.62)	41.89 (10.79)		35.21 (10.55)	39.91 (9.63)		35.09 (11.21)	36.91 (9.21)		32.69 (11.11)	38.67 (10.56)		32.78 (10.30)	37.98 (11.21)		31.24 (11.21)	37.60 (11.11)		31.72 (11.55)	6.45 (4.45 to 8.45)		<.001
STAI-Y2^c^	42.21 (8.09)	36.71 (8.64)	42.97 (9.91)		36.96 (8.68)	40.62 (9.70)		34.83 (10.50)	40.07 (9.78)		33.87 (10.65)	40.30 (10.99)		33.79 (10.58)	40.20 (10.63)		32.93 (10.13)	39.96 (10.57)		33.45 (10.84)	6.66 (4.70 to 8.62)		<.001
PIBQ^d^	3.61 (3.20)	3.46 (3.40)	3.39 (3.05)		3.12 (3.06)	3.19 (2.84)		2.94 (2.86)	4.01 (3.89)		3.44 (3.93)	3.64 (4.82)		3.39 (4.41)	3.22 (3.20)		3.12 (3.41)	2.93 (2.87)		2.64 (2.76)	0.60 (0.11 to 1.10)		.02
PES^e^	29.00 (5.28)	29.97 (5.23)	27.72 (5.14)		28.95 (5.06)	29.99 (5.06)		30.07 (4.86)	31.74 (4.63)		31.28 (5.53)	32.11 (4.80)		31.77 (5.06)	32.22 (4.49)		32.29 (4.85)	32.63 (4.35)		32.59 (4.68)	−0.99 (−1.99 to 0.02)		.05
PSSP^f^	13.34 (4.53)	12.04 (4.65)	14.57 (5.19)		12.91 (5.23)	14.03 (5.43)		11.87 (5.09)	13.78 (5.82)		11.29 (4.74)	13.52 (5.38)		10.92 (4.94)	13.27 (5.90)		10.83 (4.95)	13.14 (5.67)		10.84 (4.78)	2.17 (1.31 to 3.02)		<.001
WPBL^g^	80.12 (12.74)	85.53 (11.79)	79.13 (14.54)		82.29 (13.44)	82.35 (15.29)		83.28 (15.05)	84.75 (14.97)		87.01 (11.37)	86.01 (10.11)		87.60 (12.52)	86.40 (11.30)		86.95 (12.44)	87.97 (9.17)		88.67 (10.42)	−4.38 (−6.71 to −2.05)		<.001

^a^EPDS: Edinburgh Postnatal Depression Scale.

^b^STAI-Y1: State-Trait Anxiety Inventory state anxiety.

^c^STAI-Y2: State-Trait Anxiety Inventory trait anxiety.

^d^PIBQ: Parent-to-Infant Bonding Questionnaire.

^e^PES: Parenting Efficacy Scale.

^f^PSSP: Perceived Social Support for Parenting.

^g^WPBL: What Being the Parent of a Baby is Like.

## Discussion

### Principal Findings

This study examined the effectiveness of SPA on parental outcomes during the perinatal period. Most of the parents participating in this study were Malay and Chinese, and most of them worked full time. The most popular features of the SPA were the knowledge base and expert advice column. Owing to the longitudinal nature of the study, there was an attrition rate of 28.5%, which was higher than expected, because of the failure to complete follow-up questionnaires and disengagement with the SPA intervention. Furthermore, the control group had higher attrition compared with the intervention group. The lower level of social support received by parents in the control group because of the lack of SPA might have made them even more occupied with parenting responsibilities and thus less motivated to respond to follow-up messages. As the COVID-19 outbreak occurred soon after the study began in February 2020, it is possible that the COVID-19 pandemic undermined some of the positive impacts that SPA could have had on parental outcomes because of the uncertainties and unprecedented stress experienced by parents during this period. Nonetheless, the intervention group generally showed more improvements in parental outcomes than the control group.

The increase in EPDS scores from baseline to 1 month post partum indicated that parents from both groups experienced more depressive symptoms after their baby was born. This was not unexpected, as previous research has shown that mothers who scored higher on the EPDS after childbirth reported significantly poorer well-being and experienced difficulties in coping with daily responsibilities [41]. Bielawska‐Batorowicz and Kossakowska‐Petrycka [42] also found that major discrepancies between prenatal expectations and postnatal realities were significant causes of PND in men. Similarly, parents in this study might have struggled to cope with new parenting responsibilities during the postnatal period, leading them to experience greater depressive symptoms. However, the SPA intervention did not appear to have a significant impact on reducing the PND symptoms of the parents in the intervention group. This could be because of the additional stressors caused by the COVID-19 pandemic [43], which might have diminished the impact of the SPA intervention in reducing PND, as both new and experienced parents were faced with new struggles that they had not encountered before COVID-19. Joy et al [44] found that the isolation caused by safe distancing restrictions caused parents to feel less supported, and some parents also had feelings of loss as they were unable to share the joys of their newborn with other family members. In the context of Singapore and other parts of Southeast Asia, many women from all races undergo a confinement period after childbirth, where they engage in a set of postpartum practices that enhance recovery from pregnancy and labor for approximately 1 month after childbirth [45]. Many parents receive help from family members during this period with tasks such as doing household chores, cooking, and caring for the mother and infant [45]. However, isolation because COVID-19 made it difficult for parents to receive such familial and instrumental support, causing them to lose an important source of support during this period [14]. These could be the reasons that though parents had access to the various types of support, including informational and emotional support, via the SPA, they were missing the support of their loved ones, which is seen as a crucial support system after childbirth in the Southeast Asian culture.

Consistent with existing literature, this study found that mothers were more likely to exhibit possible PND symptoms, as the mean EPDS scores of mothers were significantly higher than those of fathers [46]. In this study, the graphs of EPDS scores for mothers and fathers from the intervention and control groups showed different trends. For the control group, both parents showed similar patterns in the changes in PND scores over time. This is similar to the findings of previous research, in which one parent was more likely to develop PND if their partner had PND [47]. Thus, it is possible that mothers and fathers in the control group influenced each other’s PND scores. In contrast, the fathers in the intervention group showed a steady improvement in PND symptoms until 6 months postpartum, when the EPDS scores started increasing. Thus, although having access to the SPA improved the well-being of fathers during the 6-month intervention period, this effect did not persist after the intervention ended. Most fathers did not seek support from peer volunteers as they preferred informational rather than emotional support. However, fathers also had a consistently low mean EPDS score of 5 from the fourth month postpartum onward, which may explain why the SPA intervention did not continue to reduce depressive symptoms. In contrast, the depressive symptoms of mothers in the intervention group continued to decrease from 1 month post partum to well after the intervention ceased, whereas mothers in the control group reported more depressive symptoms from 9 to 12 months postpartum. McLeish et al [48] revealed that receiving peer support helps new parents feel valued and empowered, reducing their depressive and anxiety symptoms by eliminating feelings of isolation. This would be the reason why peer support provided by the SPA intervention could have had long-term effects on improving the depressive symptoms of the mothers in the intervention group.

The anxiety levels of parents generally decreased across all time points in both groups in this study. As elaborated previously, the COVID-19 pandemic might have hindered a larger reduction in anxiety levels, as the pandemic brought new sets of difficulties that caused parents to experience more stress [49]. Parents were unable to seek instrumental help from family members and friends, which could have been a major stressor for first-time parents, and experienced parents had their own unique needs as they had to juggle even more childcare responsibilities because of the shift to home-based learning [14]. Although the findings of our study did not show any statistical significance on anxiety scores, parents in the intervention group mostly experienced slightly lower levels of anxiety compared with parents in the control group. This could be because of the reasons highlighted by the parents in our qualitative study [14] that the SPA notifications and knowledge base contents were very helpful for them, especially when it was difficult to obtain information from health care professionals during the COVID-19 period, as parents were discouraged from visiting the hospitals and antenatal classes were canceled abruptly [14].

Contrary to the hypothesis, parent-infant bonding of the parents in the intervention group was significantly poorer than the parents in the control group at 6 months post partum. This was an unexpected result; similar to other outcomes, parents received parent-infant bonding-related educational information via the SPA. Response-shift bias could have affected how the intervention group participants responded to PIBQ. Rosenman et al [50] explained that response-shift bias occurs when one’s frame of reference changes across time points, such that the intervention effect is confounded with bias recalibration. Specifically, the knowledge content, discussion forums, and frequently asked questions in the SPA intervention might have changed the parents’ understanding of how good parent-infant bonding should be, causing them to be more critical of their own experiences of bonding with their newborn. Therefore, this response-shift bias could have caused a significant difference between the PIBQ scores of the parents in the control and intervention groups. In-depth qualitative interviews should be considered in the future to obtain a better understanding of parental bonding beyond what self-report measures can offer.

This study found that the scores for parental self-efficacy and parenting satisfaction were highly similar in both the intervention and control groups. The mean PES and WPBL scores of all parents were also high, even at baseline. This negatively skewed distribution of scores may be attributed to a ceiling effect [51]. The lack of statistically significant differences between the control and intervention groups for these 2 outcomes might be because the parents already had high parental self-efficacy and satisfaction at baseline. Thus, this ceiling effect caused the SPA intervention to have a limited impact on these 2 outcomes. One reason for this ceiling effect could be that the psychoeducational content evaluated in previous studies by this study’s research team has already been adapted in these study venues. The research team has evaluated psychoeducational interventions focusing on parental self-efficacy and social support in previous studies [10,28,52] at many public health care institutions in Singapore. These public health care institutions have thus been presenting up-to-date evidence-based psychoeducational content to parents as part of standard care. This led to higher self-efficacy and satisfaction scores for the parents. Furthermore, some parents, especially those in the control group who did not have access to the SPA, were using other parenting apps. The apps that they used might have played a role in increasing their self-efficacy and perceived social support. However, this study did not collect data regarding parents’ use of other parenting apps and their awareness of the parenting information available in hospitals. Future studies should collect data regarding other informational resources used by parents to further determine the effectiveness of the intervention. Nonetheless, this is a positive finding, as parental self-efficacy and parental satisfaction are factors contributing to better parent and child well-being. Studies have found that positive self-efficacy in parents is correlated with positive attitudes toward parenting, better family role construction, higher maternal and paternal involvement, and better parental mental health [53,54]. Parent satisfaction has also been found to be correlated with marital satisfaction and a positive parent-child relationship [55]. Therefore, the parents in this study generally had high levels of self-efficacy and satisfaction.

The SPA intervention provided both informational and emotional support, both of which are important forms of social support that parents desire during the perinatal period [3,4]. Overall, parents in the intervention group felt that they received more social support than parents in the control group. This can be explained by the findings from the qualitative study that was part of this RCT, where not only did parents in the intervention group perceive more social support but they also felt more satisfied with the emotional support they received from female peer volunteers [14]. The SPA was designed to be convenient and reliable, with notifications that kept parents updated on what they could expect at each stage of pregnancy or baby development postpartum. With the support of peer volunteers, the mothers in the intervention group felt more reassured that someone was there for them. Mothers felt more well supported than fathers. This is likely because the mothers were more receptive toward the peer support offered to them, whereas the fathers were less inclined to seek peer support from the volunteers. However, further research is needed to confirm the specific types of support appreciated by parents and to evaluate the effectiveness of peer support for fathers provided by other similar fathers.

### Strengths and Limitations

Using technological advancements to our advantage, a technology-based intervention consisting of both informational and emotional support was developed to fulfill the needs of parents during the perinatal period. The SPA intervention served as a one-stop support, offering various types of pregnancy- and parenting-related content as well as assigning trained peer volunteers to improve the mental well-being of parents. The findings of this study are based on the existing literature regarding how technology-based interventions can affect parental outcomes. Gender differences in outcome scores and trends also suggest that different interventions may be needed to meet the specific and unique needs of both mothers and fathers.

Despite the ample sample size, there was still an overall attrition rate of 28.5%, which was higher than the 20% initially postulated. The long follow-up duration (up to 12 months postpartum) and the outbreak of COVID-19 might have caused this high attrition rate. Another reason for this attrition rate could be parents’ disengagement with the SPA intervention. However, this study did not obtain sufficient data on the SPA user engagement to distinguish between the 2 causes of the attrition rate. Recent literature [56] has emphasized the importance of investigating user engagement in mHealth apps to provide a better understanding of user experiences and to maintain or increase users’ activities on these apps. Thus, it is important for future studies on mHealth apps to develop extensive plans around how to investigate and build user engagement. On the basis of our experiences, future studies can build better rapport with the participants by checking in with parents more regularly, sending encouraging messages, and reminding parents to use the SPA for their parenting- and pregnancy-related concerns. Closer communication between the research team and peer volunteers can also be facilitated to provide more specialized support suited to the needs of the parents so that the intervention can be more engaging for the parents.

Another limitation of this study was that, owing to human error, there was no information on whether the parents recruited were new or experienced. This is a major limitation, as the experiences, struggles, and support needs of new and experienced parents can differ widely. Future studies need to include this information to provide a greater depth of understanding how the intervention impacts the differing needs of these parents. Such human errors occurred mainly because the majority of the study team members were clinicians who had to devote their attention to supporting their parents in clinical settings during the pandemic. Hence, the lack of regular meetings between team members has led to an increased incidence of human errors and compromised communication.

No data on the parents’ use of other parenting apps were collected. This was also a limitation of this study, as the use of these other mobile apps could have provided greater insight into the interpretation of our results. Furthermore, we believe that because of the hectic duties juggled by parents, they might forget to access the SPA for information or advice from experts and peer volunteers, despite regular notifications. As the SPA was only programmed to send regular notifications depending on the stage of pregnancy or age of the newborn, some parents might have accidentally switched off receiving those notifications, or those who received them could have been preoccupied with settling other pressing demands around parenting during the evolving COVID-19 related restrictions. It is also possible that the notifications were not received if the parents did not update the SPA when prompted. Thus, more frequent robust ways of reaching out to parents beyond regular notifications may be needed so that parents can visit the app more actively to maximize the benefits they can reap from the SPA intervention.

### Conclusions

This study examined the effects of the SPA intervention on parental outcomes. Despite the statistically insignificant effects of the SPA intervention, the mothers in the intervention group generally fared better. In particular, the parents in the intervention group felt that they received greater social support than the parents in the control group. Owing to their convenience, technology-based interventions are becoming increasingly popular, resulting in the need to develop mobile apps that are both appealing and reliable. Peer support has also been found to be a viable option for mothers to receive peer support; however, more research is needed to innovate better ways to support the mental well-being of fathers. Further improvements and evaluations such as building better rapport with parents to improve attrition rate, providing more frequent reminders for parents to access the SPA, and developing ways to provide emotional support to fathers need to be made to ensure the effectiveness of the SPA intervention in enhancing parental outcomes as well as parent and child well-being.

## Appendix

Multimedia Appendix 1 CONSORT eHealth Checklist (v 1.6.1).

## Abbreviations

CONSORT: Consolidated Standards of Reporting Trials
EPDS: Edinburgh Postnatal Depression Scale
mHealth: mobile health
PES: Parenting Efficacy Scale
PIBQ: Parent-to-Infant Bonding Questionnaire
PND: postnatal depression
PSSP: Perceived Social Support for Parenting
RCT: randomized controlled trial
SPA: Supportive Parenting App
STAI: State-Trait Anxiety Inventory
WPBL: What Being the Parent of a Baby is Like
